# Cognitive impairment in first psychotic episodes: The role of cannabis

**DOI:** 10.1192/j.eurpsy.2021.2150

**Published:** 2021-08-13

**Authors:** S. Boi, B. Sanz-Aranguez, M.T. González Salvador, R. Gutierrez Labrador, C.M. Martín Ruíz, L. Caballero, R. De Arce Cordón

**Affiliations:** 1 Psychiatry, Hospital Universitario Puerta de Hierro de Majadahonda, Madrid, Spain; 2 Psychiatry, HOSPITAL UNIVERSITARIO PUERTA DE HIERRO MAJADAHONDA, MADRID, Spain; 3 Psychiatry, Hospital Infanta Sofía, San Sebastián de los Reyes, Spain; 4 Psiquiatría, Hospital Universitario Puerta de Hierro, Majadahonda, Spain

**Keywords:** cannabis users, cognitive impairment in first-episode psychosis, psychosis, first-episode psychosis

## Abstract

**Introduction:**

The role of cannabis on cognitive impairment in first-episode psychosis (FEP) is becoming more important, with multiple investigations on this regard, with heterogeneous results due to different methodologies.

**Objectives:**

To evaluate the cognitive profile in patients who suffer a FEP, analyzing the potential effect of cannabis.

**Methods:**

We present the preliminar results of a cross-sectional case-control study about the effect of cannabis on cognition, in patients suffering a FEP. We recruited a total of 41 FEP during the last two years. We investigated the theorical differences between those who were cannabis users (FEP-Cannabis+)(n=28) and not cannabis users (FEP-Cannabis-)(n=13). We included a control group with healthy subjects who were cannabis users (Healthy-Cannabis+)(n=24).Sociodemographic and clinical questionnaire was completed. The Screening Scale to evaluate Cognitive Impairment in Psychiatry(SCIP-S) and the Cannabis Abuse Screening Test (CAST) were used. The consumption pattern was also evaluated.

**Results:**

In this study, cognitive impairment was found in FEP-Cannabis+,when compared with Healthy-Cannabis+.The most affected areas were immediate verbal learning (I-VL), delayed verbal learning (D-VL), processing speed (PS), and total score (TS). Significant differences were also observed in the cognitive profile of patients suffering FEP depending on their use of cannabis. FEP-Cannabis+) showed lower scores in PS, I-VL and TS.

**Figure:**
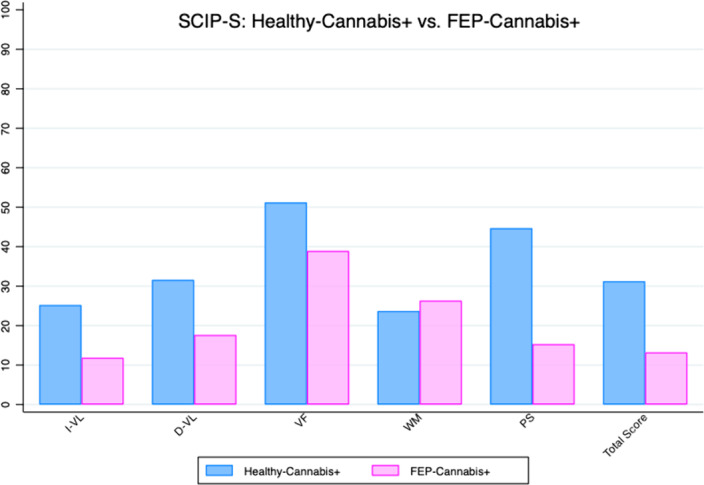

**Figure:**
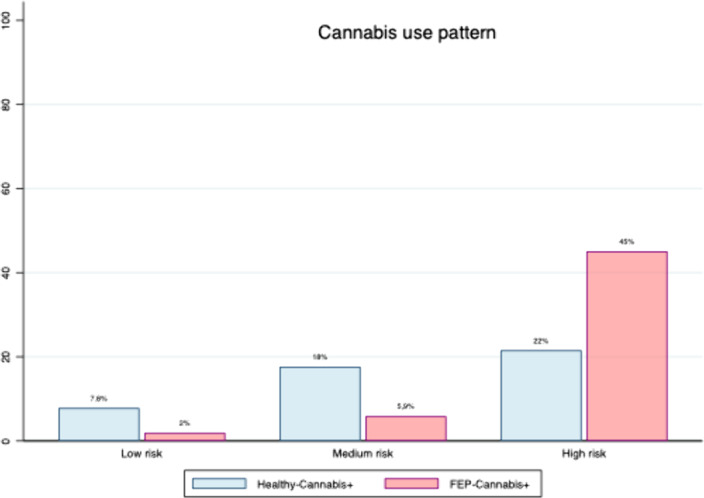

**Conclusions:**

Although several prognostic factors have been identified in FEP, to date there are no reliable markers for predicting the possible evolution of high-risk mental states to a FEP. More investigations are necessary in order to elucidate the role of cannabis in the cognitive impairment.

**Disclosure:**

No significant relationships.

